# Remote Sensing Inversion of Water Quality Grades Using a Stacked Generalization Approach

**DOI:** 10.3390/s24206716

**Published:** 2024-10-18

**Authors:** Ziqi Zhao, Luhe Wan, Lei Wang, Lina Che

**Affiliations:** 1 College of Geographical Sciences, Harbin Normal University, Harbin 150025, China; 18745388297@163.com (Z.Z.); wanglei@hljit.edu.cn (L.W.); chelinana@126.com (L.C.); 2 Heilongjiang Wuyiling Wetland Ecosystem National Observation and Research Station, Yichun 153000, China; 3 Wuyiling Wetland Ecosystem Field Scientific Observation and Research Station, Yichun 150059, China

**Keywords:** water pollution, water quality grades classification, machine learning (ML), landsat-8, remote sensing (RS), sentinel-2

## Abstract

Understanding water quality is crucial for environmental management and policy formulation. However, existing methods for assessing water quality are often unable to fully integrate with multi-source remote sensing data. This study introduces a method that employs a stacking algorithm within the Google Earth Engine (GEE) for classifying water quality grades in the Songhua River Basin (SHRB). By leveraging the strengths of multiple machine learning models, the Stacked Generalization (SG) model achieved an accuracy of 91.67%, significantly enhancing classification performance compared to traditional approaches. Additionally, the analysis revealed substantial correlations between the normalized difference vegetation index (NDVI) and precipitation with water quality grades. These findings underscore the efficacy of this method for effective water quality monitoring and its implications for understanding the influence of natural factors on water pollution.

## 1. Introduction

Water pollution has become a global issue today with industrialization and population growth [1]. Therefore, establishing effective water quality monitoring and real-time water quality early warning is of great scientific value and significance for supporting the macro decision-making of relevant departments [2]. Water quality monitoring stations in many countries test and record pollutant concentrations to evaluate overall water quality levels [3,4]. Water quality classification methods using water quality grades to evaluate pollution status have become a research hotspot among experts and scholars [5,6,7,8,9,10].

Existing research has proven the feasibility of using Machine Learning (ML) models in accurately assessing and predicting water quality grades, making them efficient tools for early warnings and environmental management [11,12,13,14]. Recently, many approaches such as random forest (RF), support vector machine (SVM), k-nearest neighbor (KNN), decision tree (DT), etc., have been proposed for classifying water quality levels [15]. Water quality can be categorized into different grades to represent pollution levels and changes based on pollution indicators, allowing for the assessment of water quality through remote sensing (RS) imagery [16,17,18,19]. The literature shows that different bodies of water exhibit different characteristics at specific wavelengths [20]. These wavelengths are often used to capture the key optical properties related to the water quality parameters to obtain the water quality grades. The specific wavebands used may vary depending on the research objectives, available satellite imagery, and characteristics of the study area [21,22,23,24,25]. Alparslan et al. [26] utilized classified water quality data and extracted relevant parameters from RS images to establish a model that employs the water quality index for classification and evaluation. Nasir et al. [20] evaluated various ML models based on the Water Quality Index for water quality classification. However, previous studies often used single ML algorithms at specific sites, resulting in subjective classification rules and a limited application scope. Furthermore, previous studies using a single RS image source for water quality grade classification could not capture some local or transient water quality changes. In contrast, the use of multiple image sources is usually required to reliably identify water quality grades [20,21,22,23,24,25,26,27]. Therefore, there is a need for cost-effective tools for continuous, regional-scale water quality monitoring, facilitating the efficient prediction of water quality levels.

When providing water quality grade inversion in large areas using satellite imagery, several challenges must be addressed when using ML. Firstly, most prediction models are developed using original ML algorithms without feature optimization or integration, making them inadequate for large-scale water quality assessment. Secondly, few studies explore the impact of natural factors on water quality grades, limiting their practical applicability. Additionally, some previous studies relied solely on water quality parameters to determine water quality grades [28], further complicating the evaluation process.

In this context, we present a method for classifying water quality grades by integrating multiple ML models through a stacking algorithm based on ensemble learning. The proposed method was applied to classify water quality grades for the Heilongjiang Province section of the SHRB using Landsat-8 Operational Land Imager (OLI) and Sentinel-2 Multispectral Instrument (MSI) remote sensing images, achieving an accuracy exceeding 91%. The objectives of this study were to (1) establish datasets for training and testing machine learning algorithms, including RF, Classification and Regression Tree (CART), KNN, SVM, and Gradient Tree Boosting (GTB) within GEE, using the Sequential Forward Selection (SFS) algorithm for feature selection and integrating top-performing models into a stacking algorithm; (2) analyze the influence of natural factors (precipitation, soil nitrogen, vegetation) on water quality grades using the Spearman rank correlation coefficient; and (3) implement long-term monitoring of river water quality grade using multi-source RS data, reducing dependence on water quality parameters.

## 2. Materials and Methods

### 2.1. Framework of Water Quality Evaluation

**Figure 1:**
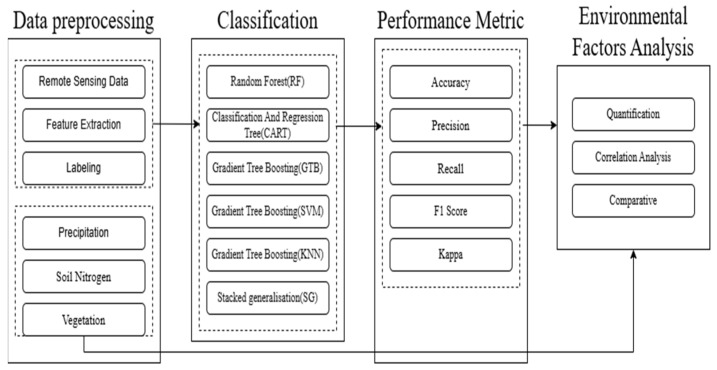
Methodology for water quality classification using RS imagery. The methodology includes data preprocessing, classification, performance metrics, and environmental factors analysis.

In this study, we utilized both individual Landsat-8 OLI and Sentinel-2 MSI imagery, as well as fused RS imagery combining data from both sources. This approach allowed us to leverage the strengths of each dataset and enhance the overall analysis. The methodology involved several steps: data preprocessing, classification, performance metrics, and environmental factors analysis (Figure 1).

Since optical satellite imagery may be contaminated by clouds and shadows, leading to missing values in the imagery, a preprocessing step should be performed to handle missing values, extract features, and label the data to make it suitable for machine learning classifiers. At this stage, the relevant environmental factors should also be prepared as the basis for subsequent analysis. The next phase is classification, which is the core part of this study. In addition to using five basic machine learning classification models, we also constructed a Stacked Generalization (SG) model, combining the predictions of multiple classifiers to improve overall performance. To the best of our knowledge, few studies have applied a combination of multiple classifiers to multisource and multitemporal imagery for water quality classification. After classification, the next step is to evaluate the performance of the models using various metrics. The final step involves analyzing environmental factors based on the classified data. The following subsections explain each component of the framework and its application.

### 2.2. Study Area and Dataset Details

**Figure 2:**
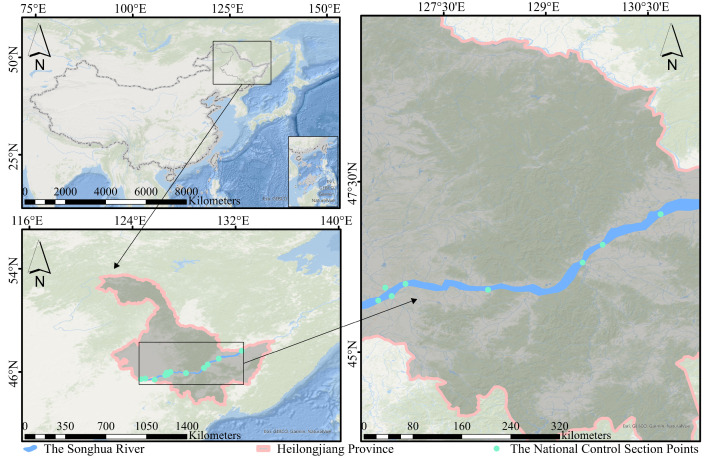
An overview map of the SHRB in the study area and the spatial distribution of national control points (indicated by green dots) where regular parameter measurements are conducted.

The study area (Figure 2) is one of the seven major river basins in China, with a drainage area of 556,800 square km [29]. It is situated in a climate zone characterized by a north-temperate monsoon, and the rainfall varies significantly throughout the year [30]. These national control points, selected for their widespread distribution and representative characteristics, serve as reliable data sources for water quality grade inversion. The water quality grade in the SHRB exhibited improvement from 1990 to 2018, with a specific emphasis on the comparative analysis between stage I (1990–2005) and stage II (2005–2018) [28].

The satellite data used in this study were the atmospherically corrected Landsat-8 and Sentinel-2 datasets on the GEE platform (https://earthengine.google.com), accessed on 1 May 2022. The parameters of each band of the OLI remote sensing images from the Landsat-8 dataset and the MSI remote sensing images from the Sentinel-2 dataset are presented in Table 1. To improve the accuracy of water quality monitoring, a histogram matching technique was applied to fuse the spectral bands of Landsat-8 and Sentinel-2, ensuring consistency between the datasets. The input variables for the water quality inversion model included texture parameters from the Gray Level Cooccurrence Matrix (GLCM), the Normalized Difference Water Index (NDWI), and remote sensing reflectance data from the OLI and MSI sensors. Ground truth data were collected during ground surveys conducted from May to July 2022 at national control section collection points from the National Surface Water Quality Automatic Monitoring Data Release System (http://www.cnemc.cn/) accessed on 1 May 2022, which are sites where parameters are measured regularly in the field. These data were used to create datasets, totaling 200 points across different classes. The points were randomly divided into training and testing sets with a 2:8 ratio.

We address the problem of water quality grade classification for the SHRB in Heilongjiang by using multi-source RS images. The evaluation of surface water environmental quality is based on achieving the designated water body function category by selecting the corresponding category standards and performing a single-factor evaluation. These standards apply to surface water bodies with specific uses, including rivers, lakes, canals, channels, and reservoirs within China. In this study, water quality was determined by measuring the concentration values of each monitored chemical indicator, following the national standard GB3838-2002. According to this standard, water quality is classified into six grades: I, II, III, IV, V, and worse than V. Previous studies have demonstrated that this standard is highly suitable for evaluating China′s water environment [32]. Several representative national control section points are listed (Table 2). The main water quality parameters corresponding to different water quality grades are listed, with the standard values for each water quality grade provided in parentheses [23].

### 2.3. Classification

ML algorithms have been widely used in water quality classification to provide accurate and efficient analysis. Recently, some ML classifiers have been integrated into GEE′s application programming interface (API), allowing users to efficiently analyze large amounts of geospatial data online. In this paper, we established RF, CART, SVM, GTB, KNN, and SG models in the GEE. The SG model integrates RF, SVM, and CART as base classifiers using a stacking algorithm to extract diverse features and minimize classification errors. Through K-fold cross-validation, these classifiers are trained on different subsets of the dataset to ensure robust predictions. Their outputs are put into a new feature set to be used as input for a linear regression meta-classifier. This approach allows the meta-classifier to learn the strengths of each base model, assigning optimal weights to their predictions. By reducing overfitting, handling feature dependencies, and leveraging the complementary strengths of each model, the SG model provides more accurate and generalizable water quality predictions than individual classifiers. However, the inclusion of GLCM texture features, spectral reflectance bands, and NDWI in the model inputs, sourced partly from Landsat-8 and partly from Sentinel-2 fused data, necessitates the need for feature selection due to its dependency and redundancy of spectral features.

In our experiment, to optimize our model′s performance, we utilized SFS in conjunction with grid search. This approach involved a forward search for feature selection and a grid search for parameter optimization, thereby achieving an optimal balance between model complexity and predictive accuracy. SFS optimizes model accuracy and reduces overfitting by systematically eliminating less significant features. All datasets and code are available for online access on GitHub (https://github.com/zzq18846/GEE_water_quality/tree/64acaa19d729b5441484e07ac94c698b07b9cc32), accessed on 24 September 2024.

### 2.4. Prediction Performance Assessment

During the training process, cross-validation in statistical analysis methods was used to evaluate the model. Following training, the six models were tested using the training and testing datasets. The confusion matrix helps visualize the probability and degree of correct or incorrect classification across various water quality levels. It is used to calculate key parameters like accuracy, precision, recall, F1-score, and the Kappa coefficient, which are essential for evaluating the performance of different water quality prediction models [33]. The calculation formulas for these evaluation indices are as follows:

(1) $$Accuracy=\frac{TP+TN}{TP+TN+FP+FN}$$

(2) $$Precision=\frac{TP}{TP+FP}$$

(3) $$Recall=\frac{TP}{TP+FN}$$

(4) $$\mathrm{F1 score}=\frac{2\cdot Recall\cdot Precision}{Recall+Precision}$$

(5) $$Kappa=\frac{p_{o}-p_{e}}{1-p_{e}},\;p_{e}=\frac{a_{1\cdot }b_{1}+a_{2\cdot }b_{2}+\ldots +a_{c\cdot }b_{c}}{n\cdot n}$$

where TP represents true positive; TN represents true negative; FP represents false positive; FN represents false negative; $p_{o}$ represents the observed agreement, and $p_{e}$ represents the chance agreement. $a_{i}$ is the sum of the elements in the ith row of the confusion matrix, $b_{i}$ is the sum of the elements in the ith column of the confusion matrix, c is the number of classes, and *n* is the total number of observations. The aforementioned evaluation process was implemented in Python (version 3.8.5).

## 3. Result

### 3.1. Optimal ML Algorithm Selection

Six ML models were established for the effective classification and determination of water quality levels. To enhance classification accuracy, the SG model employed a linear model as a meta-learner to integrate three other models with superior performance. This ensemble learning approach aimed to improve the overall predictive capability of the system.

**Figure 3:**
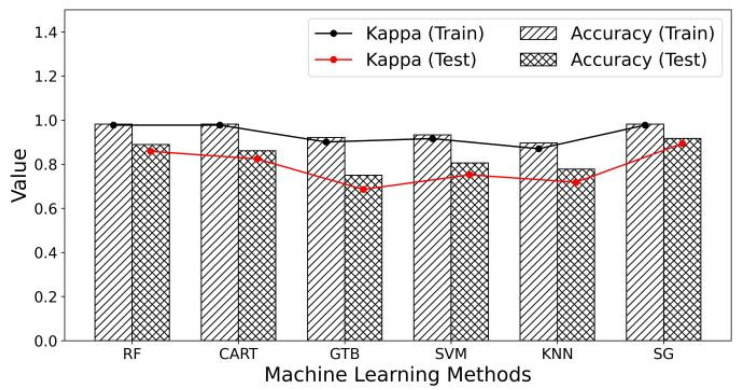
Model accuracy and Kappa score.

Figure 3 shows the accuracy and Kappa coefficient of each algorithm on the training and testing datasets. In the training set, the SG model performs similarly to the RF and CART models but significantly outperforms other algorithms, achieving an accuracy of 98.17% and a Kappa coefficient of 0.98. On the testing set, the SG model continued to perform well, with an accuracy of 91.67% and a Kappa coefficient of 0.89.

### 3.2. Comparison of Prediction Results

In this study, we used measured water quality grade data from national control sections in the Songhua River Basin (SHRB), Heilongjiang Province, Northest China, to train and test six predictive models. These models were then employed to predict the water quality levels in the SHRB. Table 3 presents the results of the performance measurements with six classifiers on both the training and testing sets. The evaluation indices, Accuracy, Precision, Recall, F1 Score, and Kappa, for SG (0.92, 0.92, 0.92, 0.92, and 0.90, respectively) were the highest among the five algorithms.

**Figure 4:**
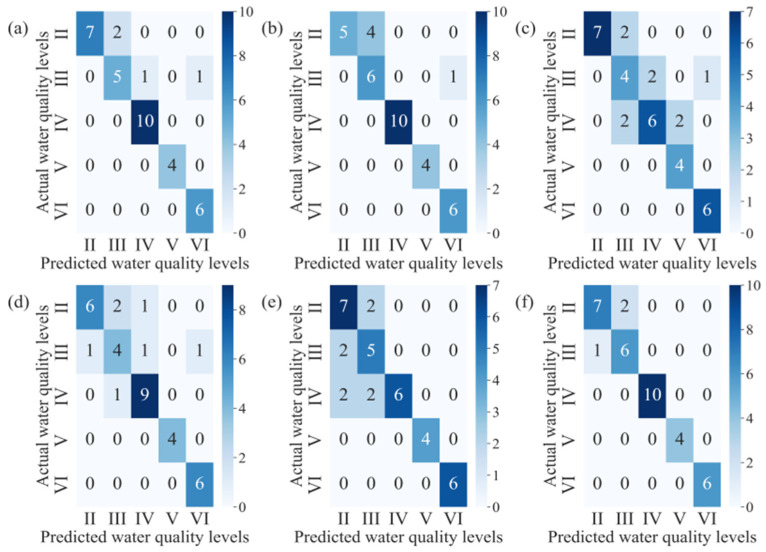
Confusion matrices of prediction results for six ML models: (**a**) RF; (**b**) CART; (**c**) GTB; (**d**) SVM; (**e**) KNN; and (**f**) SG.

Figure 4 is plotted to more effectively present the classification results of the predictive models. In the figure, the confusion matrix illustrating the prediction results is presented for the six ML methods. The cumulative numbers in the rows of the confusion matrices represent the actual water quality levels in the prediction set, while the numbers in the columns represent the predicted labels. It is important to note that the results presented in the figure are based on a sample of 200 data points, specifically illustrating 36 samples. With only four samples for water quality grade V, achieving high sensitivity from the prediction models is challenging. Among the six ML algorithms, the SG model, which utilizes the RF as a base classifier, demonstrated the highest accuracy in water quality classification, with only three misclassifications across all predicted water quality levels. This explains the observed performance differences. Therefore, the SG model is highly suitable for future water quality predictions in the SHRB.

### 3.3. Inversion of Water Quality Grade

After applying the inversion model to the Landsat-8 remote sensing images, the inversion results for various water quality grades were obtained. Finally, the classified water quality grades were visually presented, effectively illustrating the spatial distribution patterns of different water quality levels within the study area.

**Figure 5:**
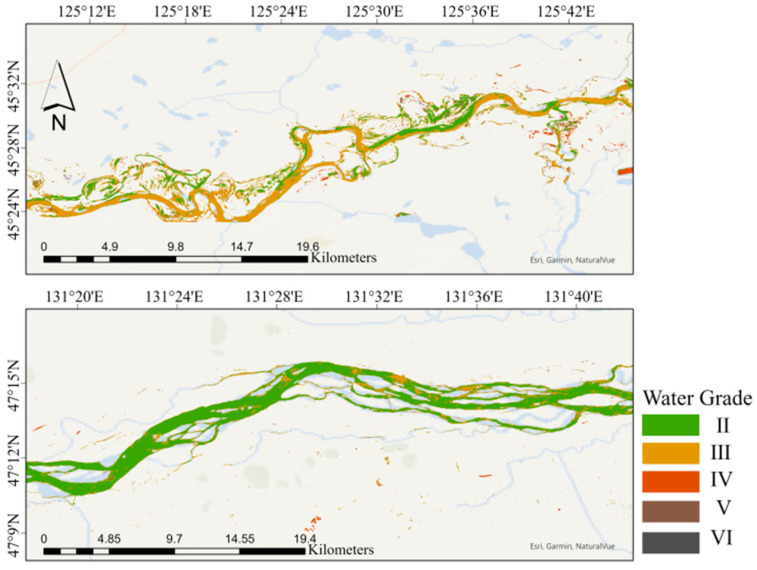
The inversion results of water quality grades in parts of the SHRB within Heilongjiang Province.

Figure 5 shows the inversion results of water quality grades for parts of the SHRB within Heilongjiang Province. This figure illustrates the spatial distribution of water quality across the region, providing critical insights for environmental monitoring and management. The absence of Class I water in the inversion results indicates that the water quality in this area does not meet the strictest standards for drinking water sources or other high-quality uses.

## 4. Discussion

### 4.1. Environmental Factors Analysis

After generating the water quality grade map of the study area referencing the water quality, which is influenced by many environmental factors as summarized by Chaudhry et al. [1], we analyzed the impacts of precipitation, soil nitrogen, and vegetation on water quality. In general, obtaining datasets directly related to factors causing water pollution within watersheds is challenging. The datasets of relevant environmental factors in this paper were downloaded from GEE and processed using Python. This approach leverages the vast amounts of satellite data available on GEE, overcoming traditional limitations of data availability and accessibility.

Table 4 shows the statistical analysis results of environmental factors concerning the different grades of water quality. After preparing the datasets of driving factors, we calculated the Spearman correlation coefficients to explore linkages between these factors and water quality grade values. Subsequently, we aimed to better understand their influence on water quality grades. The Spearman correlation coefficients indicate positive associations between normalized difference vegetation index (NDVI) values (0.2518) and precipitation (0.2592) with water quality grades, while showing a negative correlation (−0.1818) between nitrogen and water quality grades. Xu et al. [34] have demonstrated the contribution of NDVI to water quality, and previous studies [35] have indicated that precipitation can influence inland water quality by moderating land use characteristics. Our findings in this paper further support the impact of vegetation coverage, as represented by NDVI, and precipitation on water quality grades.

### 4.2. Limitations and Future Research

Current studies lack detailed information on the inversion of surface water quality grades across different seasons. The data used in this research were predominantly collected during the summer, which is insufficient for capturing the seasonal variability of water quality. Seasonal changes are significant, and the factors contributing to water quality often differ depending on the season [34]. In light of these limitations, we recognize the importance of validating our method on a larger dataset that encompasses multiple seasons to fully account for the dynamic nature of natural processes. While our current dataset was supplemented through multi-source remote sensing fusion where water quality grades were inferred primarily from spectral information, this approach may still yield inaccuracies in the extraction process. Previous studies [33,35] have demonstrated that supervised classification techniques perform well in water quality assessment and monitoring. Therefore, to address the current dataset′s limitations, future research will focus on expanding the dataset to include seasonal variations and leveraging big data. By introducing additional variables and employing more sophisticated modeling techniques, we aim to significantly enhance the accuracy of water quality grade inversions. This future direction will not only bolster our findings but also contribute to a more comprehensive understanding of water quality dynamics throughout the year.

In summary, this study found that the SG model is most suitable for the inversion of water quality grades in the Heilongjiang Province section of the Songhua River Basin, and this finding has practical significance for water quality management and decision-making. Additionally, applying machine learning techniques in the GEE for water quality assessment has the potential to change water quality management practices. It supports a data-driven, scalable, and cost-effective approach to monitoring and protecting water resources, contributing to the region′s sustainable development and environmental management.

## 5. Conclusions

Water quality grade is considered an indicator of the water quality condition. In this study, we proposed a method for water quality grade classification that integrates multiple ML models using a stacking algorithm based on ensemble learning. We used Landsat-8 OLI and Sentinel-2 MSI images of the SHRB in Heilongjiang Province. By employing the stacking algorithm, we integrated the results of three effective machine learning models: RF, CART, and SVM. This ensemble learning approach allowed us to achieve more accurate water quality grade classification. Additionally, we used the SFS algorithm for feature selection. In general, the SG classification model exhibited the best performance in water quality grades, with an accuracy rate of 91.67%. In addition, our quantitative analysis of environmental factors revealed positive associations between NDVI values and precipitation with water quality grades. Specifically, the Spearman correlation coefficients for NDVI and precipitation were 0.2518 and 0.2592, respectively. These findings emphasize the critical role of environmental variables in shaping water quality, thereby informing government policies for the restoration and management of the SHRB.

Notably, this study provides key insights into the application of ensemble learning methods for water quality assessment, aiding in the development of more effective environmental management strategies. The results are significant because ensemble learning models can be adapted to various geographic regions and water systems, making valuable contributions to broader environmental monitoring and water resource management efforts. In particular, this research offers a more dynamic understanding of how water systems respond to external pressures, helping to develop adaptive and resilient water management frameworks on a larger scale. Future work can focus on integrating additional environmental variables and seasonal effects to further improve the robustness of water quality predictions and their applicability across different regions. Expanding the scope to include these factors will enable deeper insights into water system responses, supporting more adaptive, data-driven approaches to regional water resource management.

## Figures and Tables

**Table 1:** Band specifications for Landsat-8 and Sentinel-2 dataset images [31].

Landsat-8			Sentinel-2		
Band	Wavelength (nm)	Resolution (m)	Band	Wavelength (nm)	Resolution (m)
SR_B1 (Aerosols)	435–451	30	B1 (Aerosols)	443.9	60
SR_B2 (Blue)	452–512	30	B2 (Blue)	496.6	10
SR_B3 (Green)	533–590	30	B3 (Green)	560	10
SR_B4 (Red)	636–673	30	B4 (Red)	664.5	10
			B5 (Red Edge 1)	703.9	20
			B6 (Red Edge 2)	740.2	20
			B7 (Red Edge 3)	782.5	20
SR_B5 (NIR)	851–879	30	B8 (NIR)	835.1	10
			B8A (Red Edge 4)	864.8	20
			B9 (Water vapor)	945	60
SR_B6 (SWIR-1)	1566–1651	30	B11 (SWIR-1)	1613.7	20
SR_B7 (SWIR-2)	2107–2294	30	B12 (SWIR-2)	2202.4	20

**Table 2:** The selected national control section points in different water grades in the study area (values in brackets are standard line in the grade).

Points	Grade	Title 3		
		NH_3_-N	TP	TN
Zhushuntun	I	0.025 (0.15)	0.005 (0.02)	0.34 (0.02)
Dadingzishan	II	0.268 (0.5)	0.048 (0.1)	3.29 (0.5)
Miaojia	III	0.723 (1.0)	0.068 (0.2)	3.90 (1.0)
Jiayin	IV	0.025 (1.5)	0.016 (0.3)	0.41 (1.5)
Humashang	V	0.06 (2.0)	0.77 (0.4)	0.76 (2.0)
Tongjiang	VI	0.025 (2.0)	0.025 (0.4)	0.96 (2.0)

**Table 3:** Results of the performance measurements with six classifiers on both the training and testing sets.

Dataset	Algorithms	Accuracy, %	Precision, %	Recall, %	F1 Score	Kappa
Train	RF	98.17	98.33	98.18	0.98	0.97
	CART	98.17	98.23	98.18	0.98	0.97
	SVM	93.29	93.44	93.11	0.93	0.92
	GTB	92.07	91.93	91.68	0.92	0.90
	KNN	89.63	91.37	88.97	0.89	0.87
	SG	98.17	98.17	98.12	0.98	0.98
Test	RF	88.89	89.53	89.84	0.89	0.86
	CART	86.11	89.84	88.25	0.86	0.82
	SVM	80.56	80.66	78.98	0.80	0.75
	GTB	75.00	77.25	82.76	0.75	0.69
	KNN	77.78	82.27	81.84	0.78	0.72
	SG	91.67	92.01	92.70	0.92	0.89

**Table 4:** Descriptive statistics of environmental factors by water quality grade.

Factor	Grade	Max	Min	Mean	Median
Precipitation (nm)	II	3.47	2.29	2.92	2.95
	III	3.57	2.33	2.85	2.59
	IV	3.36	3.11	3.24	3.24
	V	3.21	3.21	3.21	3.21
Nitrogen (g/kg)	II	0.24	0.00	0.07	0.01
	III	0.15	0.00	0.07	0.08
	IV	0.00	0.00	0.00	0.00
	V	0.01	0.01	0.01	0.01
NDVI	II	−0.17	−0.57	−0.46	−0.55
	III	−0.11	−0.33	−0.22	−0.22
	IV	−0.20	−0.82	−0.51	−0.51
	V	−0.20	−0.20	−0.20	−0.20

## Data Availability

Data available on request due to restrictions.
